# Adaptive optimal control of entangled qubits

**DOI:** 10.1126/sciadv.abq4244

**Published:** 2022-12-07

**Authors:** David L. Goodwin, Pranav Singh, Mohammadali Foroozandeh

**Affiliations:** ^1^ Chemistry Research Laboratory, University of Oxford, Mansfield Road, Oxford OX1 3TA, UK.; ^2^ Department of Mathematical Sciences, University of Bath, Bath BA2 7AY, UK.

## Abstract

Developing fast, robust, and accurate methods for optimal control of quantum systems comprising interacting particles is one of the most active areas of current science. Although a valuable repository of algorithms is available for numerical applications in quantum control, the high computational cost is somewhat overlooked. Here, we present a fast and accurate optimal control algorithm for systems of interacting qubits, QOALA (quantum optimal control by adaptive low-cost algorithm), which is predicted to offer O(*M*^2^) speedup for an *M*-qubit system, compared to the state-of-the-art exact methods, without compromising overall accuracy of the optimal solution. The method is general and compatible with diverse Hamiltonian structures. The proposed approach uses inexpensive low-accuracy approximations of propagators far from the optimum, adaptively switching to higher accuracy, higher-cost propagators when approaching the optimum. In addition, the utilization of analytical Lie algebraic derivatives that do not require computationally expensive matrix exponential brings even better performance.

## Teaser

QOALA offers fast optimal control of entangled qubits by adaptive combination of low-cost and high-accuracy solvers.

## INTRODUCTION

Driving quantum spins systems to a desired target state via optimal control theory (*1*) has been widely applied to a range of areas including nuclear magnetic resonance (NMR) (*2*, *3*), magnetic resonance imaging (*4*, *5*), electron paramagnetic resonance (*6*, *7*), quantum error correction and quantum information registers (*8*, *9*), cold atoms (*10*, *11*), terahertz technologies (*12*, *13*), control of trapped ions (*14*, *15*), and nitrogen vacancy centers in diamond (*16*, *17*). Along with applications in measurement science, algorithmic and numerical developments of optimal control methods remain active and challenging, with examples including geometric (*18*, *19*) and adiabatic (*20*, *21*) optimal control, GRAPE (gradient ascent pulse engineering) (*22*, *23*) and Krotov (*24*, *25*) algorithms, tensor product approach for large quantum systems (*26*), and optimal control over approximate control landscapes (*27*).

A crucial ingredient in these optimal control applications is a numerical method for computing the dynamics of spin systems, which is used for computing the objective function at each iteration of an optimization algorithm. A common feature in most numerical methods is that a uniformly and highly accurate method is used throughout the optimization process when, for the most substantial part, the optimization cannot benefit from the provided accuracy and hence suffers from the computational burden without much gain. This problem becomes more evident and cumbersome, especially in the context of large, multiparticle systems, and therefore more important to address.

Here, we present a general and highly flexible approach for solving optimal control problems for systems of entangled qubits in a computationally efficient manner without sacrificing the desired accuracy. In an optimization process, computationally inexpensive methods for computing spin dynamics can be used far from the optimum, where loss of accuracy is less crucial and where the most iterations in a numerical optimization routine are often spent. Conversely, high-accuracy methods should only be used for computing the dynamics of quantum systems close to the optimum when high fidelity is achievable and desired. This adaptive optimal control method, QOALA (quantum optimal control by adaptive low-cost algorithm), uses a set of approximate propagators that are designed to allow variable degrees of trade-off between accuracy and computational expense and achieves significant speedup in practice. We elucidate the potential speedup of QOALA with the concrete example of a class of numerical methods called propagator splittings. However, the overall framework developed here is flexible enough to incorporate any combination of numerical methods with different cost and accuracy trade-offs.

**Fig. 1.**
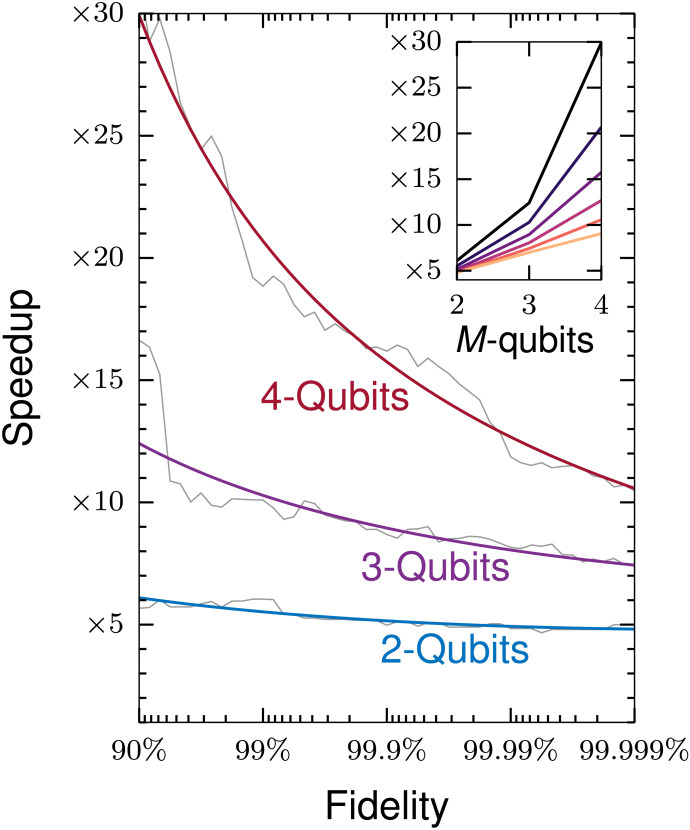
Speedup of QOALA state transfer optimal control problems. Speedup in wall-clock time achieved by using the QOALA method when compared to an exact method for state transfer optimal control problems. Smooth, thick lines are a polynomial fit to the thin lines. The inset plot shows the expected speedup, per qubit, for different fidelities from 90% (top line) to 99.9999% (bottom line).

This paper is structured as follows: In the “Formulation of optimal control problem,” “Termination criteria,” and “The adaptive procedure” sections, a brief and general theory of optimal control is presented, and the concept of adaptive approach is introduced. In the “QOALA with propagator splitting” section, the ingredients of the QOALA method including the Hamiltonian structure, propagator splitting, and computation of derivatives are presented, along with potential benchmarking strategies. Lastly, in the “Numerical demonstrations” section, demonstrations of the proposed method are presented in the context of NMR using a range of examples including state transfers and swap gates on two-, three-, and four-spin systems. In each case, the convergence and wall-clock time of the method are compared to methods (*28*–*30*) available via the versatile software package Spinach (*31*). It is demonstrated that the proposed adaptive framework consistently outperforms available methods. Relative speedup of QOALA compared to an exact method is shown in Fig. 1.

## RESULTS

### Formulation of optimal control problem

The state of a system with *M* spins at time *t* is described by a density matrix ρ(*t*), and its dynamics are governed by the Liouville–von Neumann equation

$$\frac{\partial \rho (t)}{\partial t}=-iH(t)\rho (t),\;\rho (t_{0})=\rho_{0}$$ (1)

where ρ_0_ is the initial state of the system at time *t*_0_, and $H=1⊗H-H^{⊤}⊗1$ is the time-dependent Hamiltonian superoperator of the system. The solution of Eq. 1 can be expressed as

ρ(t)=U(t)ρ(0) (2)

Formally, the propagator **U**(*t*) is a time-ordered exponential of −iH denoted as

$$U(t)=Te^{-i\int_{0}^{t}H(t^{\prime })dt^{\prime }}$$ (3)

In optimal control applications, the Hamiltonian, H(t), depends on some control parameters, θ, and optimal choices of these parameters are sought, which can either (i) drive the state of the system ρ(*T*) at a specified time *T* to a desirable target ϱ or (ii) ensure that the propagator **U**(*T*) implements a desirable propagator **U**_target_. To highlight the dependence on θ, we often write H(*t*; θ), **U**(*t*; θ), and ρ(*t*; θ) instead of H(*t*), **U**(*t*), and ρ(*t*), respectively, where normally, *t* ∈ [0, *T*]. Further, we assume that H(*t*; θ) and **U**(*t*; θ) are represented by *N* × *N* matrices.

The optimal control problem is expressed in terms of maximizing an objective function

F[θ)=f(U(T;θ)] (4)

where *f* is a continuously differentiable real-valued functional of propagator matrices, i.e.,

$$f\in C(C^{N\times N};R)$$ (5)

Here, we are particularly concerned with cases where the value of the objective at the optimal parameters

$$\theta^{*}=\underset{\theta }{\mathrm{argmax}}F(\theta )$$ (6)

is known a priori. Without loss of generality, we assume that, after suitable scaling of *f*, the objective F is able to achieve the maximum possible value of 1

$$F(\theta^{*})=1$$ (7)

A large class of objective functions F of this form that appear in the context of optimal control of spins and gate design are fidelity functions. For instance

f(X)=Re[Tr(ϱ†Xρ0)] (8)

leads to the fidelity functional

F(θ)=Re{Tr[ϱ†U(T;θ)ρ0]}=Re{Tr[ϱ†ρ(T;θ)]}∈[0,1] (9)

which measures the overlap of the quantum state ρ(*T*; θ) at a specified time *t* = *T*, with a target state ϱ. Here, both ρ_0_ and ϱ are assumed to be improper [i.e., trace-free, Tr(ρ) = 0], normalized (‖ρ‖_2_ = 1), and Hermitian (ρ^†^ = ρ) density matrices. Functionals such as Eq. 8, where an initial state ρ_0_ appears, lead to fidelity functions that are used in the context of state-to-state transfers, i.e., case (i).

Here, normalization is only assumed so that value at the optima is 1, i.e., to satisfy Eq. 9. Note that the requirement of trace-free density matrices only applies to the fidelity functional described in Eq. 9, and the overall approach applies equally to fidelity functions of proper density matrices (i.e., non–trace-free density matrices).

The functional

f(X)=Re[Tr(Utarget†X)] (10)

leads to the fidelity

F(θ)=Re{Tr[Utarget†U(T;θ)]} (11)

which does not depend on initial state ρ_0_ and can be used where an effective design of a specified propagator **U**_target_ (a desired unitary gate, for instance) is required, i.e., case (ii). Note that, instead of working with the real value of the trace in Eqs. 8 and 10, i.e., applying the function Re( · ), we can also apply any other continuously differentiable function, e.g., absolute value squared, ∣ · ∣^2^.

Fidelity functions measure the similarity between mixed quantum states. A very wide range of fidelity functions exist, among them notably the Uhlmann-Jozsa fidelity (*32*), which satisfy the fidelity axioms to different extents. We refer the reader to (*33*) for a more detailed discussion of the appropriateness of various fidelity measures. For the purposes of this manuscript, however, all objective functions F satisfying Eqs. 4 to 7 will be considered valid fidelity functions that can be maximized using the proposed approach.

The fidelity functions can be maximized using gradient-based optimization schemes, where one needs the gradient of the fidelity function F. We compute the gradient using the chain rule

$$\frac{\partial F(\theta )}{\partial \theta }=Df\;[U(T;\theta )]\frac{\partial U(T;\theta )}{\partial \theta }$$ (12)

which further requires computation of the gradient of the propagator **U**(*T*; θ). Here, **D***f*(*X*) is the Fréchet derivative of *f* at *X*, which acts linearly on *Y*, and **D***f*(*X*)*Y* quantifies the rate of change in the direction *Y*. Equation 12 can be generalized further to use Gateaux derivatives (or directional derivatives) (*34*, *35*), where instead of **D***f*(*X*)*Y*, we write **D***f*(*X*; *Y*) or **D***_Y_f*(*X*).

For instance, the action of the Fréchet derivative of the functional in Eq. 8 is

Df(X)Y=Re[Tr(ϱ†Yρ0)]

so that the gradient of the fidelity Eq. 9 is

∂F(θ)∂θ=Re[Tr(ϱ†∂U(T;θ)∂θρ0)]=f(∂U(T;θ)∂θ) (13)

The exact propagator **U**(*T*; θ) (and hence its derivatives) are not available, since an analytical solution of Eq. 3 is not available except in very specialized and restrictive circumstances. Instead, in practice, we rely on numerical methods for solving Eq. 3.

We assume that a family of numerical solvers S_(1)_, S_(2)_, …, S_(*L*)_ to approximate the propagator **U**(*T*; θ) is available and arranged in increasing accuracy and cost, i.e., we assume that the solver $S_{(\ell +1)}$ is more accurate and computationally more expensive than $\$S_{\left(\ell \right)}\$$. Here, $\$S_{\left(\ell \right)}\$$ is a (θ-dependent) linear map from the initial state ρ_0_ to the state at time *T*

$$\rho^{\left(\ell \right)}(T;\theta )=S_{\left(\ell \right)}(\theta )\rho_{0}$$

and it produces an approximation $\$\rho^{\left(\ell \right)}\$$(*T*; θ) to ρ(*T*; θ), the true solution of Eq. 1.

The use of a particular propagator $\$S_{\left(\ell \right)}\$$ naturally leads to the computation of an approximate value of the fidelity function

$$F_{\left(\ell \right)}(\theta ):=f\;[S_{\left(\ell \right)}(\theta )]\;\approx \;f\;[U(T;\theta )]=F(\theta )$$

When a range of numerical solvers with different cost and accuracy trade-offs are available, a natural question to ask is: What is the best choice of solver? On the one hand, the most inexpensive solver S_(1)_ makes the optimization fast. However, it also leads to a less accurate approximation to the fidelity, F_(1)_(θ). This becomes particularly important in applications where high fidelities (approaching 1) are feasible and desired, and using low-accuracy approximations limits the fidelity achievable. In such cases, the most accurate solver S_(*L*)_ seems appealing. However, such a solver typically comes with a high computational cost. The use of such an accurate but costly solver seems less justified in the initial stages of optimization where the objective is very far from the optimal value.

Here, we propose an adaptive procedure where inexpensive solvers with low accuracy are used far from the optimum, and high accuracy solvers with large computational costs are used only closer to the optimum θ^*^. The framework requires the following ingredients: (i) a set of numerical solvers S_(1)_, S_(2)_, …, S_(*L*)_ with different cost and accuracy trade-offs, (ii) a method for computing gradients of these solvers with respect to θ for approximation of the fidelity gradient Eq. 12, and (iii) a low-cost, adaptive strategy to switch from a solver $\$S_{\left(\ell \right)}\$$ to a more accurate solver $\$S_{(\ell +1)}\$$, which includes an error estimation of the computed fidelity $\$F_{\left(\ell \right)}\$$ and a measure of proximity to the optimum θ^*^.

### Termination criteria

In theory, a nonlinear optimization process is terminated when the distance of the *j*th iterate θ^(*j*)^ from the optimum θ^∗^ becomes sufficiently small. In practice, however, because θ^∗^ is not available a priori, we estimate‖θ^∗^ − θ^(*j*−1)^‖ by ‖θ^(*j*)^ − θ^(*j*−1)^‖ and terminate iterations when

$$\|\theta^{\left(j\right)}-\theta^{\left(j-1\right)}\|\;\leq \;{\mathrm{tol}}_{\theta }$$ (14)

for some user-defined tolerance tol_θ_.

Another criterion is motivated by the fact that, at critical points (including, but not limited to, the optimum), the gradient vanishes, e.g., ∇_θ_F(θ^∗^) = 0. Again, some user-defined tolerance tol*_g_* is used to asses this criterion as

$$\left\|\nabla_{\theta }F[\theta^{\left(j\right)}]\right\|\;\leq \;{\mathrm{tol}}_{g}$$ (15)

The last criterion to consider is the difference of the value of the objective F at the previous iterate θ^(*j* − 1)^ from the optimal value $F(\theta^{*})$

$$|F(\theta^{*})-F[\theta^{\left(j\right)}]|$$ (16)

However, we know that when a system is fully controllable, given a set of parameters, a fidelity of 1 can be achieved, i.e., F(θ^∗^) = 1, and therefore, for some user-defined tolerance tol*_F_*

$$|1-F[\theta^{\left(j\right)}]|\;\leq \;{\mathrm{tol}}_{F}$$ (17)

can be used alongside Eqs. 14 and 15 as a termination criterion. Note that even if we do not know whether a fidelity of 1 is achievable in a particular application, in quantum optimal control, we are guaranteed that F ≤ 1 (*36*) for suitably normalized initial and target states. Thus, Eq. 17 does not lead to unnecessarily early termination and should be used alongside Eqs. 14 and 15.

### The adaptive procedure

An additional and important aspect in an adaptive process is the numerical error in the approximation of the objective F. We can, at best, rely on F_(*L*)_, generated using the most accurate numerical solver S_(*L*)_ available to us, but we would like to use the least expensive solvers S_(1)_, wherever possible. Here, we consider the true fidelity F[θ^(*j*)^], a fidelity measure that would results from exact calculation methods, and $\$F_{\left(\ell \right)}\$$[θ^(*j*)^], a fidelity when the numerical solver $\$S_{\left(\ell \right)}\$$ is being used in the *j*th iteration. For ease of notation, we have suppressed the dependence on the parameters θ in this section. Using Eqs. 16 and 17, we can construct a general system of inequalities for the adaptive procedure

$$|1-F|\;\leq \;|1-F_{\left(\ell \right)}|+|F_{\left(\ell \right)}-F|\;\leq \;(1+\kappa_{F})|1-F_{\left(\ell \right)}|\;\leq \;{\mathrm{tol}}_{F}$$ (18)

where κ*_F_* ∈ (0,1) is a user-defined parameter. This system of inequalities enforces the termination criteria such that

$$|1-F_{\left(\ell \right)}|\;\leq \;\frac{{\mathrm{tol}}_{F}}{1+\kappa_{F}}$$ (19)

and ensures that

$$|F_{\left(\ell \right)}-F|\;\leq \;\kappa_{F}|1-F_{\left(\ell \right)}|$$ (20)

The true fidelity  also satisfies the termination criterion Eq. 17. Note that κ*_F_* in Eq. 20 guarantees that at all times, during the optimization procedure, we keep the numerical error several times smaller than ∣1 − $\$F_{\left(\ell \right)}\$$, which measures the distance from optimal value [assuming F(θ^∗^) = 1].

As we approach the optimal value, we need to use more accurate solvers. When Eq. 20 is violated, the optimizer switches to a more accurate solver $\$S_{(\ell +1)}\$$. From the left side of Eq. 20, the error of the fidelity approximation $\$F_{\left(\ell \right)}\$$ using the solver $\$S_{\left(\ell \right)}\$$ should be computed

$$\varepsilon^{\left(\ell \right)}:=|F_{\left(\ell \right)}-F|$$

Because the true fidelity F is not available, we use a more accurate scheme for approximating F

$$\varepsilon^{\left(\ell_{1},\ell_{2}\right)}:=|F_{\left(\ell_{1}\right)}-F_{\left(\ell_{2}\right)}|=1-\mathrm{Tr}\left\{[\rho^{\left(\ell_{1}\right)}{]}^{†}\rho^{\left(\ell_{2}\right)}\right\},\;\ell_{2}>\ell_{1}$$

where $\$\rho^{\left(\ell \right)}\$$ = $\$\rho^{\left(\ell \right)}\$$(*t_f _* ; θ) = $\$S_{\left(\ell \right)}\$$(ρ_0_; θ). The most inexpensive estimate is given by

$$\varepsilon^{\left(\ell ,\ell +1\right)}:=|F_{\left(\ell \right)}-F_{\left(\ell +1\right)}|$$ (21)

The computation of $\$\varepsilon^{\left(\ell ,\ell +1\right)}\$$ as in Eq. 21 involves both $\$S_{\left(\ell \right)}\$$ and $\$S_{(\ell +1)}\$$ and therefore is more than twice as expensive as using $\$S_{\left(\ell \right)}\$$ alone. A simple way to mitigate this problem is to only make the decision to switch from $\$S_{\left(\ell \right)}\$$ to $\$S_{(\ell +1)}\$$ once every 5 to 10 iterations, which reduces the overall burden of computing $\$\varepsilon^{\left(\ell ,\ell +1\right)}\$$. However, the gap between tests should not be too large to avoid the fidelity deviating from the true fidelity. Although other methods like defect-based error estimators (*37*) can provide a highly accurate estimate of the error in $\$S_{\left(\ell 1\right)}\$$ without the use of a more accurate scheme $\$S_{\left(\ell 2\right)}(\ell_{2}>\ell_{1})\$$ and can be easily incorporated in our framework, as our current application does not require a very high accuracy of error estimation, we restrict the present implementation to the estimator presented in Eq. 21.

### QOALA with propagator splitting

We start with presenting the total Hamiltonian in terms of interaction ($H_{\mathrm{in}}$) and single-spin ($H_{\mathrm{ss}}$) sub-Hamiltonians

$$H=H_{\mathrm{ss}}+H_{\mathrm{in}}$$ (22)

Therefore −*i*δ*t*H = A+B such that

$$A=-i\delta tH_{\mathrm{in}},\;B=-i\delta tH_{\mathrm{ss}}$$ (23)

where δ*t* is a small change in *t*, derived from the differential in Eq. 3.

The Hamiltonian for an isolated (noninteracting) spin can be written as

$$h=a\sigma_{x}+b\sigma_{y}+c\sigma_{z}$$ (24)

where σ_α_, α ∈ {*x*, *y*, *z*} are Pauli matrices for spin $-\frac{1}{2}$, (or Pauli matrices equivalent for spins $>\frac{1}{2}$). For *M* spins, a general form of $H_{\mathrm{ss}}$ can expressed as

$$\begin{array}{rl} H_{\mathrm{ss}} & =\sum_{k=1}^{M}\underset{M-k\;\mathrm{times}}{\underbrace{1⊗\cdots ⊗1}}⊗h_{k}⊗\underset{k-1\;\mathrm{times}}{\underbrace{1⊗\cdots ⊗1}}\\ & =\left[\overset{M}{\underset{k=1}{⨁}}h_{k}\right] \end{array}$$ (25)

Any other parts of the Hamiltonian, e.g., interactions, can also be included in $H_{\mathrm{in}}$. Thanks to utilization of superoperator formalism (Liouville space) in Eq. 1, $H_{\mathrm{in}}$ can contain additional terms such as decoherence via Lindblad or Redfield equations. While exp (A) can be computed once before the optimization due to properties of the Kronecker sum, exp (B) can be expressed as

$$\begin{array}{r} \exp(B)=\exp(-i\delta {th}_{1})⊗\exp(-i\delta {th}_{2})⊗\cdots ⊗\exp(-i\delta {th}_{M}) \end{array}$$ (26)

These decomposition and separation play a key role in the mechanism of the proposed method. In principle, one can use any set of solvers for the approximation of the propagator, ordered in ascending accuracy and cost. In this work, we restrict our attention to a range of splitting methods, which are numerical solvers that approximate *e*^A+B^ in terms of products of *e*^A^ and *e^B^*. The idea of approximating a propagator using splitting techniques is well explored in the literature (*38*–*44*). Propagator splittings of arbitrarily high-order accuracy can be derived using techniques such as Baker-Campbell-Hausdorff formula (*45*), Zassenhaus splitting (*46*), Magnus expansion (*47*), and their combinations. In what follows, a specified solver using a splitting method of order *p*, having an accuracy O(δ*t^p^*), will be denoted with a subscript S*_p_*.

**Fig. 2.**
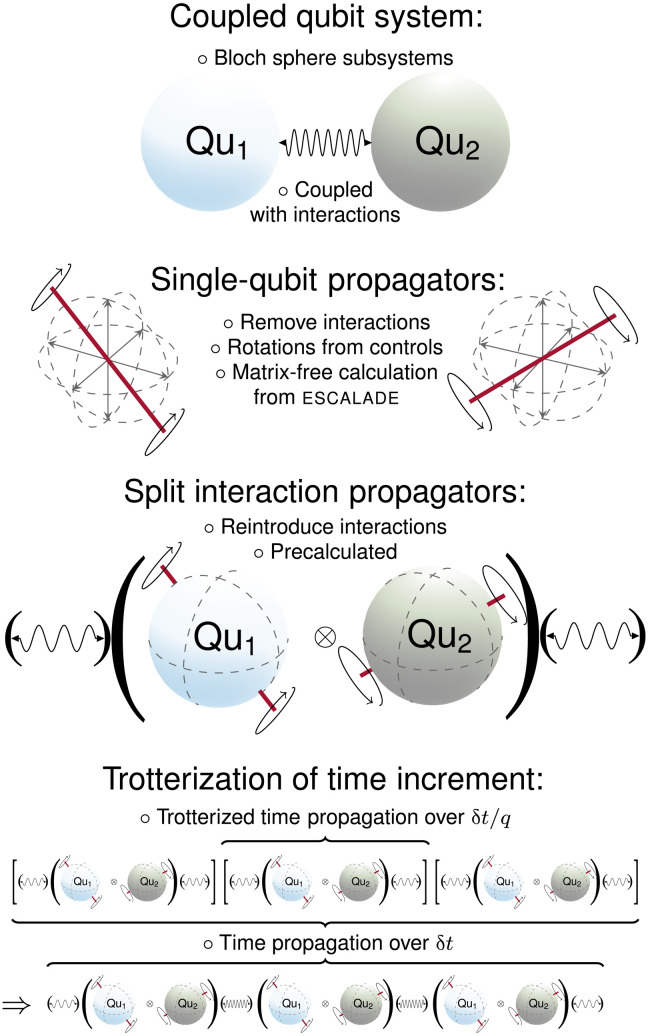
Pictorial schematics of time propagator splitting and Trotterization. A schematic diagram of a coupled two-qubit system, an angle-axis representation of ESCALADE (efficient spin control using analytical Lie algebraic derivatives) (*49*), a pictorial representation of second-order operator splitting, and Trotterization of time slices with *q* = 3. This corresponds to S_2,3_ in Table 1. The final image shows a Trotterized, split operator, time propagation with the leftmost and rightmost split coupling of the operator splitting method, allowable for S_2,*q*_, S_3,*q*_, and S_4,*q*_ in Table 1, combined into one exponential.

Moreover, any splitting method S*_p_* can be combined with Trotterization (*38*), which divides a small time slice of duration δ*t* into *q* equal, smaller, time slices of durations $\frac{\delta t}{q}$ for improved numerical accuracy, to generate a larger group of solvers

$$S_{\;p,q}(\delta t)={\left[S_{\;p}\left(\frac{\delta t}{q}\right)\right]}^{q}+O\left(\frac{\delta t^{\;p+1}}{q^{\;p+1}}\right)$$ (27)

where the Trotter number *q* is now also included as a subscript in the solver, S_*p*,*q*_. Splitting methods used as solvers in the present work are summarized in Table 1, with a schematic diagram outlining second-order splitting, *p* = 2, with Trotter number *q* = 3 shown in Fig. 2.

**Table 1.** Summary of Trotterized splitting methods as solvers to approximate $e^{A+B}$. A pictorial representation of S_2,3_ is shown in Fig. 2. The computational cost, O_cost_, is of a forward time propagation to obtain **U**(*T*; θ) for a fidelity calculation in Eq. 4.

S	Formula	O _acc._	O _cost_	Reference
*S*_1, *q*_	${\left(e^{\frac{1}{q}A}e^{\frac{1}{q}B}\right)}^{q}$	$\frac{\delta t}{q}$	2*q*	(*38*)
*S*_2, *q*_	${\left(e^{\frac{1}{2q}A}e^{\frac{1}{q}B}e^{\frac{1}{2q}A}\right)}^{q}$	$\frac{\delta t^{2}}{q^{2}}$	2*q* + 1	(*39*, *40*)
*S*_3, *q*_	${\left(e^{\frac{{\bar{a}}_{1}}{q}A}e^{\frac{b_{1}}{q}B}e^{\frac{{\bar{a}}_{2}}{q}A}e^{\frac{b_{2}}{q}B}e^{\frac{a_{2}}{q}A}e^{\frac{b_{1}}{q}B}e^{\frac{a_{1}}{q}A}\right)}^{q}$	$\frac{\delta t^{3}}{q^{3}}$	6*q* + 1	(*44*)*
*S*_4, *q*_	${\left(e^{\frac{a_{1}}{q}A}e^{\frac{b_{1}}{q}B}e^{\frac{a_{2}}{q}A}e^{\frac{b_{2}}{q}B}e^{\frac{a_{3}}{q}A}e^{\frac{b_{3}}{q}B}e^{\frac{a_{4}}{q}A}e^{\frac{b_{3}}{q}B}e^{\frac{a_{3}}{q}A}e^{\frac{b_{2}}{q}B}e^{\frac{a_{2}}{q}A}e^{\frac{b_{1}}{q}B}e^{\frac{a_{1}}{q}A}\right)}^{q}$	$\frac{\delta t^{4}}{q^{4}}$	12*q* + 1	(*41*) †

*$\begin{array}{c} a_{1}=\frac{13}{126}-i\frac{\sqrt{\frac{59}{2}}}{63},\;a_{2}=\frac{25}{63}+i\frac{5\sqrt{\frac{59}{2}}}{126},\;b_{1}=\frac{3}{10},\;\mathrm{and}\;b_{2}=\frac{2}{5}\\ a_{1}=0.0792036964311957,b_{1}=0.209515106613362 \end{array}$

†$\begin{array}{c} a_{2}=0.353172906049774,b_{2}=-0.143851773179818\\ a_{3}=-0.0420650803577195,b_{3}=\frac{1}{2}-b_{1}-b_{2},\;\mathrm{and}\\ a_{4}=1-2(a_{1}+a_{2}+a_{3}) \end{array}$

**Fig. 3.**
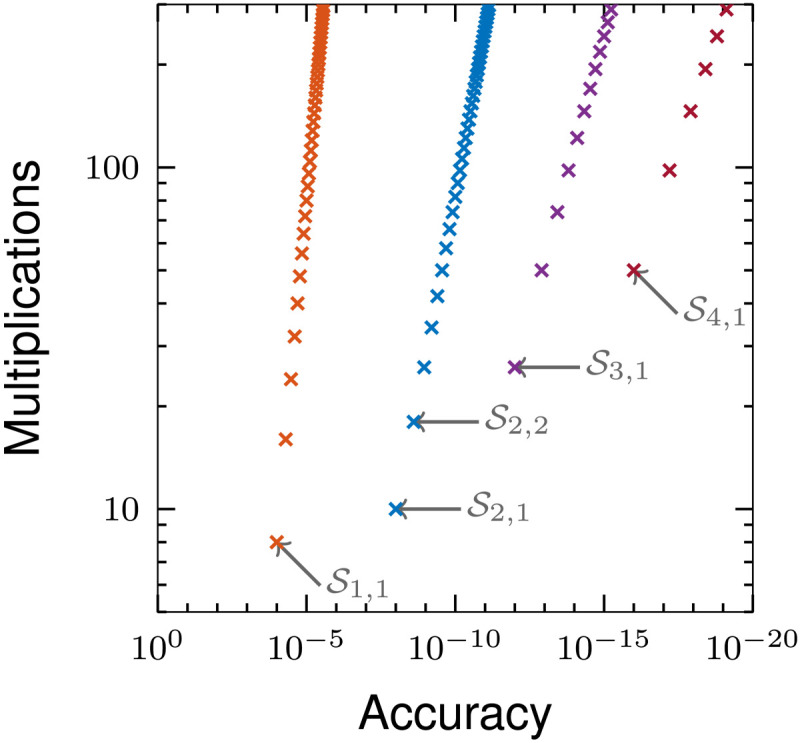
Graph showing the computational cost of different Trotterized time propagator splitting methods. The plot shows the number of multiplications per time slice required of a fidelity and gradient calculation for a desired accuracy, for δ*t* = 0.1 ms and four controls, as a measure of the computational cost of the set of solvers presented in Table 1; arrows and S_*p*,*q*_ terms represent switching points between members of the solver set.

The accuracy of each solver is known, and since the dimensions of the Hamiltonian do not change in the course of the optimization, we can consider the number of matrix-vector multiplications as a measure for cost. Using these known attributed accuracy and cost, we can obtain a generic graph of cost versus accuracy for our set of solvers (Fig. 3).

Let us recall the definition of fidelity in Eq. 4

F(θ)=f[U(T;θ)]∈[0,1]

which is the objective function to be minimized in the optimization.

Although the scope of the QOALA method is not limited to the GRAPE algorithm (*22*), here, it is presented in the context of the GRAPE method, where the time-dependent control pulses are generally presented as piecewise constant over a small time interval δ*t*

$$\theta_{a,k}(t)\to [\theta_{a,k,1}\;\theta_{a,k,2}\;\cdots \;\theta_{a,k,N}]$$

with *a* ∈ {x, y} controlling the *k*th spin. For notational convenience, the explicit time interval dependence of θ_*a*,*k*,*n*_(δ*t*) has been dropped. In this context, the effective propagator over the total pulsing time duration, *T* = *N*δ*t*, is given by

$$U(T;\theta )=U_{\mathrm{tot}}:=U_{N}U_{N-1}\cdots U_{2}U_{1},\;\mathrm{with}\;U_{n}=e^{-i(\delta t)H(\theta_{n})}$$ (28)

where θ*_n_* is a general control parameter solely affecting the *n*th propagator, **U***_n_*, and the shorthand θ*_n_* = θ_*a*,*k*,*n*_ is valid for any *a* ∈ {x, y} and *k* ∈ {1, …, *M*}. Note that for pulses that are not piecewise constant, the decomposition Eq. 28 does not hold exactly. However, similar decompositions can be obtained for arbitrary pulses using the Magnus expansion (*47*), for instance.

The gradient of the true fidelity, i.e., the derivative of the fidelity at every time point *n* with respect to a discrete control, θ*_n_*, can be written as

$$\frac{\partial F(\theta )}{\partial \theta_{n}}=Df\;(U_{\mathrm{tot}})\frac{\partial U_{tot}}{\partial \theta_{n}}$$ (29)

with

$$\frac{\partial U_{\mathrm{tot}}}{\partial \theta_{n}}=U_{N}\cdots U_{n+1}\frac{\partial U_{n}}{\partial \theta_{n}}U_{n-1}\cdots U_{1}$$ (30)

However, because of computational costs, we never compute the true fidelity Eq. 4 or its gradient Eq. 29. The central idea presented here relies on the approximation of $U_{n}=e^{-i(\delta t)H(\theta_{n})}$ using a solver $\$S_{\left(\ell \right)}\$$_,*n*_ and of the propagator **U**(*T*; θ) = **U**_tot_ by S_(𝓁)_

$$S_{\left(\ell \right)}:=S_{(\ell ),N}\;S_{(\ell ),N-1}\cdots S_{(\ell ),2}\;S_{(\ell ),1},\;\mathrm{with}\;S_{(\ell ),n}\approx U_{n}$$ (31)

Thus, instead of Eq. 4, we compute the approximate fidelity

$$F_{\left(\ell \right)}(\theta )=f\;[S_{\left(\ell \right)}(\theta )]$$ (32)

and the exact gradient of the approximate fidelity Eq. 32

$$\frac{\partial F_{\left(\ell \right)}(\theta )}{\partial \theta_{n}}=Df\;[S_{\left(\ell \right)}(\theta )]\frac{\partial S_{\left(\ell \right)}(\theta )}{\partial \theta_{n}}$$ (33)

where

$$\frac{\partial S_{\left(\ell \right)}(\theta )}{\partial \theta_{n}}=S_{(\ell ),N}\cdots S_{(\ell ),n+1}\;\frac{\partial S_{(\ell ),n}}{\partial \theta_{n}}\;S_{(\ell ),n-1}\cdots S_{(\ell ),1}$$ (34)

As a concrete example of approximate solvers, $\$S_{\left(\ell \right)}\$$, we consider the use of the splitting propagators introduced in Table 1. The *n*th propagator **U***_n_* is approximated by a split propagator, S_*p*,*q*_(θ*_n_*) = [S*_p_*(θ*_n_*)]*^q^*. For ease of notation, we drop the dependency on θ*_n_*. Assuming an odd number of multiplications, 2*P* + 1, in a general solver, S*_p_* = **S**_2*P*+1_**S**_2*P*_⋯**S**_2_**S**_1_ (making a distinction between the solver, S*_p_*, and one of its constituent matrix exponentials, **S**), and by considering that A and B appear in odd and even **S** terms, respectively (without loss of generality, as presented in Table 1), i.e.,

$$S_{2j}=e^{b_{2j}B},\;S_{2j+1}=e^{a_{2j+1}A}$$ (35)

and given the fact that $\frac{\partial A}{\partial \theta_{n}}=0$, $\frac{\partial B}{\partial \theta_{n}}\neq 0$, and $\left[\frac{\partial B}{\partial \theta_{n}},B\right]\neq 0$, for a general solver, S_*p*,*q*_, with Trotter number *q*, we have

$$\frac{\partial S_{\;p,q}}{\partial \theta_{n}}=\sum_{i=1}^{q}{\left(S_{p}\right)}^{i-1}\frac{\partial S_{p}}{\partial \theta_{n}}{\left(S_{p}\right)}^{q-i}$$ (36)

where, using S*_p_* = **S**_2*P*+1_**S**_2*P*_⋯**S**_2_**S**_1_

$$\frac{\partial S_{p}}{\partial \theta_{n}}=\sum_{\;j=1}^{P}\left[(\prod_{i=2P+1}^{2j+1}S_{i})\frac{\partial S_{2j}}{\partial \theta_{n}}(\prod_{i=2j-1}^{1}S_{i})\right]$$ (37)

$\frac{\partial S_{2j}}{\partial \theta_{n}}$ can be computed using finite difference (*48*) or exact (*30*) methods. While finite difference approximations of the gradient are easy to program, they can also be inaccurate, expensive, and most importantly, in the present context, unstable when the underlying numerical propagator has low accuracy (*49*, *50*). Here, taking advantage of the decomposition presented in Eq. 26, we use analytical Lie algebraic derivatives using the recent ESCALADE (efficient spin control using analytical Lie algebraic derivatives) method (*49*), which, in contrast to the auxiliary matrix method (*29*, *30*, *51*–*53*), does not require the computation of an expensive matrix exponential, resulting in an additional significant speedup.

### Numerical demonstrations

**Fig. 4.**
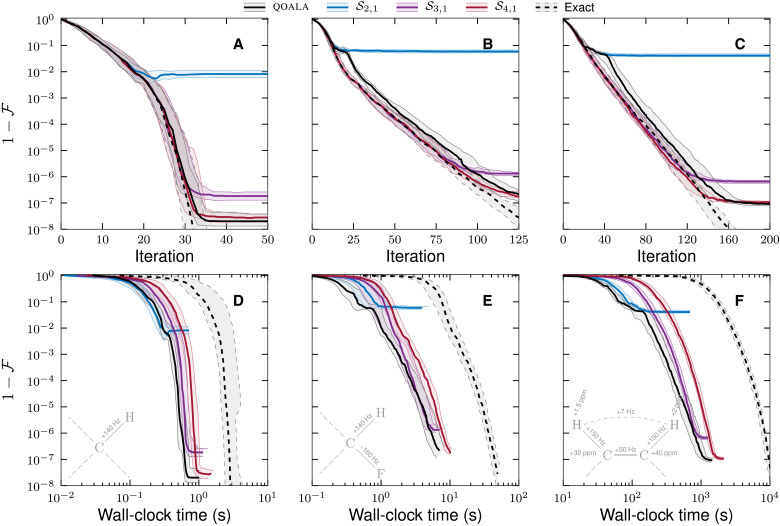
Computation time of state transfer optimal control problems. Comparison of the convergence of the two-spin (**A**), three-spin (**B**), and four-spin (**C**) state transfers with solvers ***S***_2,1_, ***S***_3,1_, and ***S***_4,1_ (solid colored) and QOALA with solver ***S***_{2,3,4},1_ (solid black) to an exact method (dashed black). The bottom plots (**D** to **F**) are the corresponding wall-clock time of computation. The two-spin system in (A) and (D) is given a pulse duration of *T* = 10 ms and a time slice width of δ*t* = 0.2 ms. The three-spin system in (B) and (E) is given a pulse duration of *T* = 22 ms and a time slice width of δ*t* = 0.2 ms. The four-spin system in (C) and (F) is given a pulse duration of *T* = 100 ms and a time slice width of δ*t* = 0.02 ms. In all plots, a central thick line is an average over 28 optimizations, and the surrounding shaded area is bound by the first and third quartiles. Adaptive splitting order selection (solid black) allows the optimization to follow a computationally inexpensive route through a convergence trajectory while achieving an eventual high fidelity. ppm, parts per million.

The state transfer problem is investigated for two different spin systems: a two-spin system, labeled system 1 in Table 2 and inset in Fig. 4D, and a three-spin system, labeled system 2 in Table 2 and inset in Fig. 4E. The two-spin and three-spin systems are both heteronuclear with 2*M* controls (*x* and *y* controls on each spin) and pulse *B*_1_ amplitude limited to 1 kHz.

**Table 2.** Spin system parameters for four different spin systems, labeled system 1 to system 3 in the first column. The second column refers to a numeric label, *k*, for each spin in the system, with the isotope of that spin in the third column. The resonance offset (chemical shift), Ω*_k_*, is shown for each spin in hertz in the fourth column. Coupling, *J_jk_*, between different spins, labeled *j* and *k*, is shown as a list in the fifth column in hertz. The static magnetic field, *B*_0_, is shown in the sixth column, and the nominal power level of the control pulses, *B*_1_, is shown in kilohertz in the seventh column. The final column shows the number of control channels. Simulations use a penalty function to ensure that control pulses do not exceed to nominal power level, **±***B*_1_, of the control pulses.

System	*k*	Isotope	Offset Ω*_k_*	Coupling, *J_jk_*	*B*_0_	*B*_1_	Controls
1	1	^1^H	Ω_1_ = 0	*J*_12_ = +140 Hz	9.4 T	1 kHz	4
	2	^13^C	Ω_2_ = 0				
2	1	^1^H	Ω_1_ = 0	*J*_12_ = +140 Hz *J*_23_ = −160 Hz	9.4 T	1 kHz	6
	2	^13^C	Ω_2_ = 0				
	3	^19^F	Ω_3_ = 0				
3	1	^1^H	Ω_1_ = −900 Hz	*J*_12_ = 150 Hz	14.1 T	10 kHz	4
	2	^13^C	Ω_2_ = −4530 Hz	*J*_23_ = 50 Hz			
	3	^13^C	Ω_3_ = −1200 Hz	*J*_34_ = 150 Hz			
	4	^1^H	Ω_4_ = −6040 Hz	*J*_14_ = 7 Hz			

Heteronuclear two-spin and three-spin systems are tasked with transferring an initial *z* magnetization from the first spin to a desired *z* magnetization at the final spin. The two-spin system is optimized over a pulse duration of *T* = 10 ms, and the three-spin system is isoptimized over *T* = 22 ms, both systems are allowed a time slice of δ*t* = 0.2 ms.

The optimal control task of a four-spin system is purposefully made more difficult: The initial state is set as the entangled multispin state defined in Eq. 45; a relaxation term is included in the uncontrollable part of the Hamiltonian. The relaxation term is calculated using Spinach (*29*, *31*, *54*, *55*) Bloch-Redfield-Wangsness relaxation theory with isotropic rotational diffusion (correlation time τ_c_ = 50 ps). The system, labeled system 3 in Table 2 and inset in Fig. 4F, is a mixture of heteronuclear and homonuclear systems and is controlled with four controls (*x* and *y* controls on each type of spin), limited to 10-kHz pulses, and optimized over a pulse duration of *T* = 100 ms with a time slice width of δ*t* = 0.02 ms.

The convergence of the two-spin system follows a typical quadratic convergence, which can be seen from the average convergence of the exact method (dashed black lines) and in Fig. 4A. The interquartile range is also shown (solid gray area bounded by thin dashed lines), with small deviations from the average indicating a predictable/robust optimization. This can be considered an easy optimal control problem. However, at the time slice width of δ*t* = 0.2 ms, the second-order splitting solver S_2,1_ fails to reach a fidelity above 99%. A similar behavior is observed for the three-spin and four-spin problems, when using the S_2,1_ solver, but with a fidelity limited to approximately 90%. The difficulty of these two optimal control problems is evident from the less than quadratic convergence of the exact method, which, after the initial damped stage of convergence, has a lower acceleration of convergence when compared to Fig. 4A.

Higher-order solvers, S_3,1_ and S_4,1_, increase the limit on achievable fidelity for all spin systems in Fig. 4 with an additional increase in computation time. Taking advantage of the short computation time of low-accuracy solvers when allowable, the QOALA algorithm switches to higher-accuracy solvers only when needed. Given a switching threshold tolerance of κ_F_ = 0.5 in Eq. 20 and a switching decision every 10 iterations, the convergence of QOALA in Fig. 4 does converge to the high fidelities of the high-accuracy solver S_4,1_ while benefiting from the time savings of the low-accuracy solvers at the beginning of the optimization. Furthermore, the average convergence trajectory of the two-spin system in Fig. 4A follows the average convergence trajectory of the exact method very closely. The first and third quartiles are also closely aligned, showing both the exact and adaptive methods converge in a predictably quadratic fashion from different, random, initial guesses. From Fig. 4 (B and C), the switch to S_3,1_ looks to have happened a little too late, following S_2,1_ for too many iterations and losing a small amount of convergence acceleration in the process. Even considering this small loss in potential time saving, all spin systems achieve an appreciable time saving compared to the exact method. Emphasizing this point further, for a four-spin system, depicted in Fig. 4F, the exact method takes more than 1.5 hours to achieve a fidelity of 99.99%, whereas QOALA takes less than 7 min to achieve the same fidelity.

**Fig. 5.**
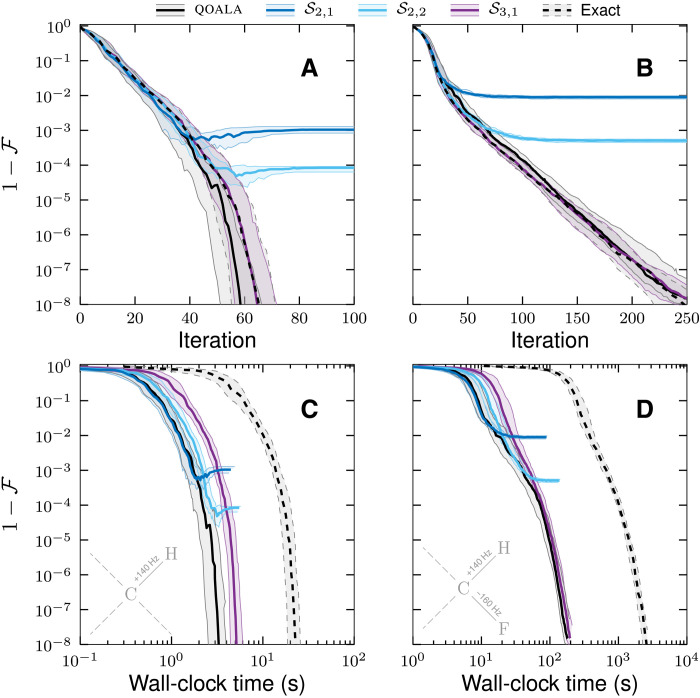
Computation time of universal swap gate optimal control problems. Comparison of the convergence of the two-spin and three-spin swap-gate with solvers ***S***_2,1_, ***S***_2,2_, and ***S***_3,1_ (solid colored) and QOALA using those same solvers adaptively (solid black) to an exact method (black dashed) (**A** and **B**) and the corresponding wall-clock time of computation (**C** and **D**). The two-spin system in (A) and (C) is given a pulse duration of *T* = 12 ms, and the three-spin system, swapping the first and third spin, in (B) and (D) is given a pulse duration of *T* = 26.4 ms. Both have a time slice width of δ*t* = 0.1 ms. In all plots, a central thick line is an average over 28 optimizations, and the surrounding shaded area is bound by the first and third quartiles. Adaptive splitting order and Trotter number selection (solid black) allow the optimization to follow an inexpensive route through a convergence trajectory: using approximate methods far from the optimum and accurate methods close to the optimum.

**Fig. 6.**
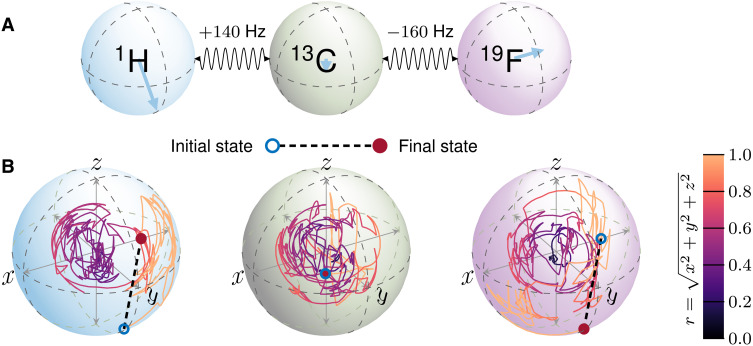
Bloch sphere trajectories for an optimal universal three-spin swap gate. Analysis of a three-spin swap gate from three-spin swap gate from Fig. 5 (B and D). (**A**) The spin system and (**B**) the optimized trajectories obtained after 250 iterations of QOALA. The initial and final states indicated in (B) show that the random states on the first and third spins are swapped, and the random initial state of the second spin is the same as the final state.

The universal swap gate problem, with the fidelity defined in Eq. 46, is investigated for two different heteronuclear spin systems: a two-spin system, labeled system 1 in Table 2 and inset in Fig. 5D. The potential time saving should be more for this type of optimization, because the efficient Krylov propagation of the exact method in state transfer problems is not appropriate here, and an explicit matrix exponential must be made in gradient calculations. In this case, the exponential of a matrix is evaluated using a Taylor series, with scaling and squaring (*35*), and truncated with a predefined tolerance of 1 ×10^−12^. Both problems have 2*M* controls (*x* and *y* controls on each spin), with pulse *B*_1_ amplitudes limited to 1 kHz. The adaptivity of the QOALA is set with a switching threshold tolerance of κ*_F_* = 0.5 (Eq. 20), a switching decision every 10 iterations. In the context of our set of examples, we observed that universal swap gate problems require slightly longer pulses than the state transfer problems. The two- and three-spin systems are optimized with *T* = 12 and 26.4 ms, respectively. An example of the effect of a three-spin swap gate is shown in Fig. 6, showing the first and third spin states swapped, and the state of the second spin is left unchanged.

In comparison to the state transfer problems in Fig. 4, the inclusion of Trotterization (S_2,2_) in Fig. 5 makes the convergence trajectory more stable by avoiding using the solver S_2,1_ for too many iterations, following the average convergence trajectory of the exact method closely in the case of the three-spin system in Fig. 5B and even finding a shortcut to convergence for the two-spin system in Fig. 5A. Again, both methods converge in a predictable fashion from initial random guesses, shown by the first and third quartiles.

**Fig. 7.**
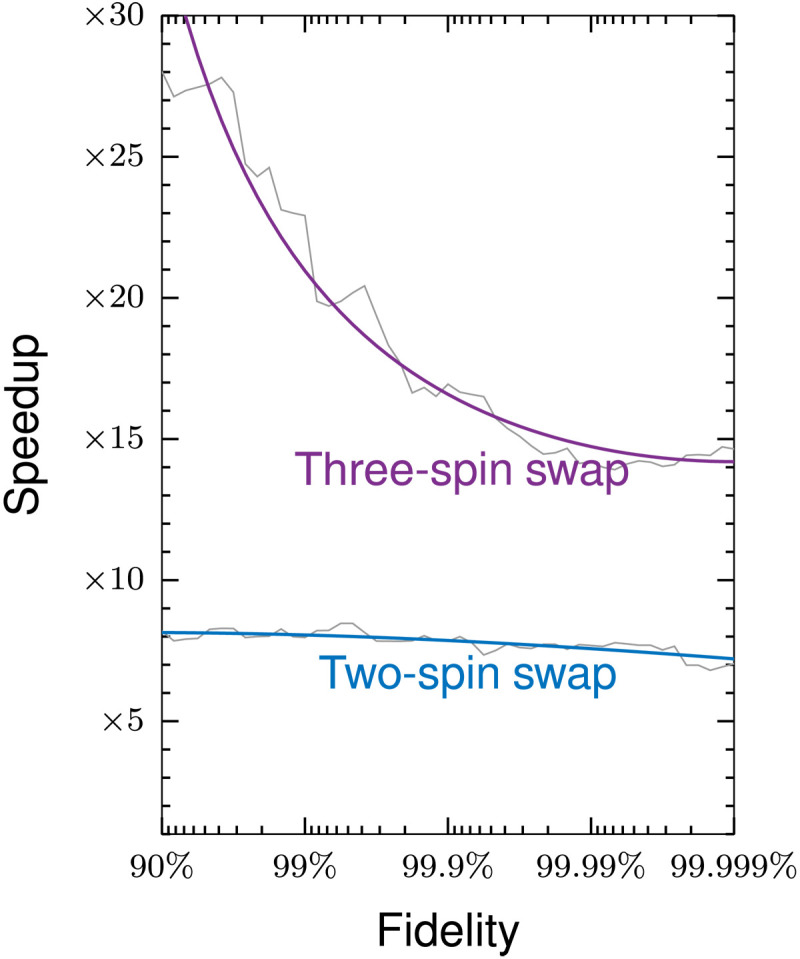
Speedup of QOALA universal gate optimal control problems. Speedup in wall-clock time achieved by using the QOALA method when compared to an exact method for universal swap gate optimizations. Smooth, thick lines are a polynomial fit to the thin lines.

Figure 7 shows the speedup achieved with the QOALA method for these two universal swap gate optimizations. It shows an even greater (an additional twofold) speedup than for state transfer problems in Fig. 1. This is because the efficiency of Krylov propagation cannot be used for propagator derivative calculations in the exact method. The slowdown from using matrix-matrix products, as opposed to efficient matrix-vector products of the state transfer problems, is inherited by both QOALA and the exact method.

## DISCUSSION

### Performance

The QOALA method starts with low-cost, low-accuracy approximations of propagators far from the optimum and adaptively switches to high-cost, high-accuracy approximations as it approaches the optimum. With a set of approximant propagators, the QOALA method offers a versatile trade-off mechanism between accuracy and computational cost.

Note that the speedup achieved with the QOALA approach compared to the exact method depends on the type of optimal control problems and the system under study, but we predict that an ∼O(*M*^2^) speedup is expected for a system consisting of *M* qubits while maintaining the accuracy of the optimal solution, as summarized in Fig. 1. High-order splitting methods are required at the highest fidelities, and the computation time of these high-order splitting methods dominate the overall computation time. The speedup tends to be O(*M*) at these high fidelities because of this domination and because there is a splitting order/Trotter number pair that gives the same accuracy as the exact method with a matrix exponential.

### Benchmarking

An alternative approach to error estimation presented in “The adaptive procedure” section is benchmarking of the solvers before the optimization. For any solver in the set of solvers, the cost and achievable accuracy is known. Precomputed benchmarking consists of identifying switch points on a cost versus accuracy plot that minimizes the overall cost while maximizing the accuracy.

If we want to apply this benchmarking strategy to the set of solvers in Table 1, then it can be seen that, with efficient matrix caching, the minimum cost of the computation of S*_p_* = **S**_2*P*+1_**S**_2*P*_⋯**S**_2_**S**_1_ and its derivative in the presence of *q*th order Trotterization is (4 + *K*)*Pq* + 2, where *K* is the number of controls. For example, the plot of Fig. 3 shows that there is no benefit in using second- and third-order solvers with Trotter numbers larger than 2 and 1, respectively.

### Concluding remarks

We presented QOALA, a fast, accurate, general, and highly flexible optimal control algorithm, for solving optimal control problems involving systems of entangled qubits in a computationally efficient manner without sacrificing the desired accuracy of the optimal solution. The results of this manuscript can be generalized in a straightforward manner to functionals that involve the values of the density matrix ρ at multiple points, as well as functionals that involve regularization and penalty terms.

## MATERIALS AND METHODS

### Liouville space

In this work, we use the spherical tensor basis (*56*) in a Liouville space, allowing a general implementation to include effects such as relaxation or decoherence. Using the Liouville-Hilbert dual relationship

$$\rho (T;\theta )=U_{\mathrm{tot}}\rho_{0}U_{\mathrm{tot}}^{†}⟺\mathrm{vec}\;[\rho (T;\theta )]=U_{\mathrm{tot}}^{*}⊗U_{\mathrm{tot}}\mathrm{vec}\;(\rho_{0}t)$$ (38)

The method is formulated in superoperator formalism

$$H\mapsto 1⊗H-H^{⊤}⊗1$$

### Hamiltonians

Although the theoretical basis of this work can be extended to any quantum systems, it is formulated and demonstrated in the context of NMR, for a spin system consisting of *M* spin −$\frac{1}{2}$ particles interacting with each other via scalar coupling *J* and under a radiofrequency pulse. The noninteracting, single-spin part of the Hamiltonian can be written as

$$H_{\mathrm{ss}}=\sum_{k=1}^{M}\theta_{x,k}I_{x,k}+\sum_{k=1}^{M}\theta_{y,k}I_{y,k}+\sum_{k=1}^{M}\Omega_{k}I_{z,k}$$ (39)

where

$$I_{a,k}=\underset{M-k\text{\textbackslash{} times}}{\underbrace{1⊗\cdots ⊗1}}⊗\sigma_{a}⊗\underset{k-1\text{\textbackslash{} times}}{\underbrace{1⊗\cdots ⊗1}},\;a\in \{x,y,z\}$$ (40)

Therefore, we can prepare this Hamiltonian for our adaptive approach by

$$H_{\mathrm{ss}}=\left[\overset{k=1}{\underset{M}{⨁}}(\theta_{x,k}\sigma_{x}+\theta_{y,k}\sigma_{y}+\Omega_{k}\sigma_{z})\right]$$ (41)

where θ*_x_* and θ*_y_* are the *x* and *y* components of the time-dependent pulse amplitudes. In the special case of *M* homonuclei, there are only two controls affecting those *M* spins, i.e., in Eq. 39, ∑*_k_*θ_*a*,*k*_**I**_*a*,*k*_ → θ*_a_*∑*_k_***I**_*a*,*k*_. The interaction Hamiltonian can be written as

$$H_{\mathrm{in}}=\sum_{\;j>k}^{M}2\pi J_{\;jk}(I_{x,j}I_{x,k}+I_{y,j}I_{y,k}+I_{z,j}I_{z,k})$$ (42)

In the case of heteronuclear coupling, the spin system can be considered weakly coupled, reducing the interaction Hamiltonian to

$$H_{\mathrm{in}}=\sum_{\;j>k}^{M}2\pi J_{\;jk}I_{z,j}I_{z,k}$$ (43)

### Auxiliary matrix method

One method to find the time propagators and analytical propagator derivatives is to use an auxiliary matrix method (*29*, *30*, *51*–*53*) for computing the matrix exponential of a block triangular matrix

$$\exp\;\left\{\left[\begin{array}{cc} A+B_{n} & C_{a,k}\\ 0 & A+B_{n} \end{array}\right]\right\}=\left[\begin{array}{cc} U_{n} & \;\frac{\partial U_{n}}{\partial \theta_{a,k,n}}\\ 0 & \;U_{n} \end{array}\right]$$ (44)

where C_*a*,*k*_ = − *i*δ*t***I**_*a*,*k*_ for a control pulse amplitude θ_*a*,*k*,*n*_ of duration δ*t*. The propagators can be recovered from one of the diagonal blocks, and the propagator derivatives with respect to θ_*a*,*k*,*n*_, in the direction of the control operator **I**_*a*,*k*_ (Eq. 40), can be extracted from the upper right block. For state transfer problems, this method should also use efficient Krylov propagation, avoiding the need to explicitly compute the matrix exponential (*57*–*59*).

### Optimal control problems

Three types of optimal control problem are compared in this work.

#### z to z transfer

The goal is to transfer an initial *z* magnetization on a spin *j* to *z* magnetization on a spin *k*: **I**_z,*j*_ → **I**_z,*k*_. Magnetization on each spin can be depicted in a simple way through Cartesian coordinates on each spin’s Bloch sphere, as in Fig. 2. Here, we use the state-to-state transfer fidelity Eq. 9, F = Re[Tr(𝜚^†^**U**_tot_ρ_0_)].

#### Entangled to z state transfer

An initial two-spin entangled state between two spins *j* and *k* asks the optimization to collapse the entanglement to a desired localized *z* magnetization on a further spin *l*: **E**^(*j*,*k*)^ → **I**_z,*l*_. The normalized, entangled, two-spin state between spins *j* and *k* is defined as

$$E^{\left(j,k\right)}=\frac{1}{2}1-\left(2I_{z,j}I_{z,k}+I_{+,j}I_{-,k}+I_{-,j}I_{+,k}\right)$$ (45)

where 1 is a unit state, a trivial but complete addition to the basis set, and **I**_±_ = **I***_x_* ± *i***I***_y_*. Once again, the fidelity used is Eq. 9, F = Re [Tr(ϱ^†^**U**_tot_ρ_0_)].

#### Universal swap gate

A universal multispin operation designed to swap the arbitrary initial magnetization between two spins, *j* and *k*, is termed as a swap gate: **I**_(*j*)_ ⇌ **I**_(*k*)_. The swap gate can be found by optimizing to a desired effective propagator, with a fidelity defined through only the effective propagator of the target

F=Re[Tr(Uswap†Utot)], (46)

where **U**_swap_ is the effective propagator of the swap operation (*60*). This fidelity function is of the form Eq. 11, and the overlap is now defined in a more general way by the Hilbert-Schmidt inner product. The real part disregards a trivial global phase that does not affect the swap gate operation.

A number of different spin systems are set out in Table 2, which are used to investigate convergence and benchmark wall-clock time of QOALA in comparison to exact calculations of propagators and propagator derivatives using the auxiliary matrix method (*29*) of Eq. 44. As mentioned above, state-to-state simulations for the exact method were performed using the efficient Spinach implementation (*31*) of Krylov propagation within MATLAB.

### Optimization strategy

All results were obtained using a workstation running CentOS 7 with a 14-core Intel Xeon W-2175 CPU at 2.50 GHz and 64 GB of RAM. All optimizations were run 28 times, from 28 different random initial pulse guesses, with their convergence trajectories and time of computation (the wall-clock time), and averaged to show typical convergence and computation behavior. The 𝓁-BFGS (limited-memory Broyden-Fletcher-Goldfarb-Shanno) gradient following optimization method is used in all cases, with the 25 most recent gradients forming a Hessian approximation (*28*). A bracketing and sectioning line search method (with cubic interpolation) calculates an appropriate step length using strong Wolfe conditions (*50*). When an adaptive method switches between solvers, a step length of 1 is selected, with no further line search at that iteration. Termination conditions of optimization are tol_θ_ = 1 × 10^−12^ in Eq. 14 and tol*_g_* = 1 × 10^−12^ in Eq. 15. All optimal control problems are given sufficient pulse duration to achieve high fidelity (*18*) and enforce a pulse amplitude spill-out penalty beyond 1 kHz (or 10 kHz for four-spin examples) (*30*), and fidelities are normalized so the globally optimal fidelity is unity. Essentially, there is the requirement that there are enough controls (enough time slices) to control the system to its desired target. This is a requirement for both an exact method and the solver-based methods, S_*p*,*q*_, presented here. Accordingly, the control problems are designed to have enough time slices to attain good convergence, but no more.
